# Temporal trends and demographic risk factors for hospital admissions due to carbon monoxide poisoning in England

**DOI:** 10.1016/j.ypmed.2020.106104

**Published:** 2020-07

**Authors:** Aina Roca-Barceló, Helen Crabbe, Rebecca Ghosh, Anna Freni-Sterrantino, Tony Fletcher, Giovanni Leonardi, Courtney Hoge, Anna L. Hansell, Frédéric B. Piel

**Affiliations:** a UK Small Area Health Statistics Unit (SAHSU), Department of Epidemiology & Biostatistics, School of Public Health, Imperial College London, London, UK; b Environmental Epidemiology Group, Centre for Radiation, Chemical and Environmental Hazards, Public Health England, Chilton, UK; c Medicines and Healthcare Products Regulatory Agency, London, UK; d National Institute for Health Research Health Protection Research Unit (NIHR HPRU) in Health Impact of Environmental Hazards, UK; e London School of Tropical Medicine & Hygiene, London, UK; f Asthma and Community Health Branch, National Center for Environmental Health, Centers for Disease Control and Prevention (CDC), USA; g Oak Ridge Institute for Science and Education, USA; h Centre for Environmental Health and Sustainability, University of Leicester, Leicester, UK

**Keywords:** Hospital Episode Statistics, Carbon monoxide, International comparison, Ethnicity, Sociodemographic status, Prevention policies, Age standardisation, Incidence risk ratio, Epidemiology

## Abstract

Unintentional non-fire related (UNFR) carbon monoxide (CO) poisoning is a preventable cause of morbidity and mortality. Epidemiological data on UNFR CO poisoning can help monitor changes in the magnitude of this burden, particularly through comparisons of multiple countries, and to identify vulnerable sub-groups of the population which may be more at risk. Here, we collected data on age- and sex- specific number of hospital admissions with a primary diagnosis of UNFR CO poisoning in England (2002–2016), aggregated to small areas, alongside area-level characteristics (i.e. deprivation, rurality and ethnicity). We analysed temporal trends using piecewise log-linear models and compared them to analogous data obtained for Canada, France, Spain and the US. We estimated age-standardized rates per 100,000 inhabitants by area-level characteristics using the WHO standard population (2000–2025). We then fitted the Besag York Mollie (BYM) model, a Bayesian hierarchical spatial model, to assess the independent effect of each area-level characteristic on the standardized risk of hospitalization. Temporal trends showed significant decreases after 2010. Decreasing trends were also observed across all countries studied, yet France had a 5-fold higher risk. Based on 3399 UNFR CO poisoning hospitalizations, we found an increased risk in areas classified as rural (0.69, 95% CrI: 0.67; 0.80), highly deprived (1.77, 95% CrI: 1.66; 2.10) or with the largest proportion of Asian (1.15, 95% CrI: 1.03; 1.49) or Black population (1.35, 95% CrI: 1.20; 1.80). Our multivariate approach provides strong evidence for the identification of vulnerable populations which can inform prevention policies and targeted interventions.

## Highlights

• Hospital admissions for carbon monoxide (CO) poisoning have decreased in England and other high-income countries.
• Striking differences in age-standardized rates per 100,000 inhabitants were observed within Europe.
• In England, the risk of UNFR CO poisoning is higher in rural areas, deprived areas, and ethnically diverse areas.
• A better understanding of national, regional and local risk factors can help informing future prevention policies.

## 1 Introduction

Carbon monoxide (CO) is an odourless, tasteless and colourless gas which can rapidly reach life-threatening levels indoors following the incomplete combustion of carbon-based fuel products including those burned in heating and cooking appliances (Ashcroft et al., 2019). Most unintentional exposures are preventable through appropriate ventilation, maintenance of burning appliances and the use of CO detectors. Nevertheless, a high proportion of household appliances are often identified as unsafe (Smollin and Olson, 2008; Penney et al., 2010; Gas Safety Register, 2016), suggesting that more prevention could be done.

Unintentional CO poisoning remains an important cause of preventable morbidity and mortality worldwide (Stearns and Sircar, 2019; Khadem-Rezaiyan and Afshari, 2016; Daoudi et al., 2011; Lavigne et al., 2015; Braubach et al., 2013; Fisher et al., 2013, Fisher et al., 2014; Yari et al., 2012). The Global Burden of Disease (GBD) added CO poisoning estimates in 2017 and reported 35,500 (95% CI: 25,700–38,800) deaths and 1462.4 (95% CI: 1,073,000; 1,613,600) disability-adjusted life years, globally for that year (Roth et al., 2018). In addition to lethal exposures, CO poisoning can have substantial short- or long-term health implications depending on concentrations inhaled, duration of exposure and physical condition (Ashcroft et al., 2019; Smollin and Olson, 2008; Knobeloch and Jackson, 1999). Morbidity studies are scarce and often limited to one small geographical area (Khadem-Rezaiyan and Afshari, 2016; Yari et al., 2012; Nazari et al., 2010; Dianat and Nazari, 2011; Wilson et al., 1998), with only a limited number reporting national estimates (Stearns and Sircar, 2019; Lavigne et al., 2015; Ghosh et al., 2016). The health and economic burden of CO poisoning are therefore likely to be underestimates.

In England, unintentional non-fire related (UNFR) is the most common cause of CO poisoning, with an average of 25 deaths per year being reported between 2015 and 2016 by the Office of National Statistics (ONS) (Office for National Statistics, 2017; Health and Safety Executive, 2019) and a hospitalization rate of 0.49/100,000 between 2001 and 2010 (Wilson et al., 1998; Ghosh et al., 2016; Clarke et al., 2012). Risks of UNFR CO poisoning vary across countries (Braubach et al., 2013), over time (Bosch, 2013; McCann et al., 2013) and by population characteristics (Ralston et al., 2012). Although monitoring temporal trends and comparing countries can provide valuable information about risk factors and the efficacy of interventions, few epidemiological studies have addressed these questions. Ghosh et al conducted a spatial-temporal analysis of hospital admissions due to UNFR CO poisoning in England only (Ghosh et al., 2016). Braubach and colleagues compared mortality data across 28 of the MemberStates of the WHO European Region between 1980 and 2008 (Braubach et al., 2013), excluding UK.

Finally, there is some evidence that specific sub-groups of the population are at higher risk than the general population (Ralston et al., 2012; National Energy Action, 2017). A pilot study funded by the Gas Safety Trust (GST), a UK gas safety research charity, on low-income households across England and Wales, identified the elderly and those living in rural areas and under fuel poverty as those at higher risk of living with unsafe CO levels in their households (National Energy Action, 2017), yet this has not been explored thoroughly using nationally representative routine health data. Identifying vulnerable population sub-groups can help inform public health strategies, including awareness campaigns, prevention plans or budget allocation.

Herein, we analysed 15 years (2002–2016) of small-area hospital admission data for England to: i) describe temporal trends in England, and compare them with those reported in other high-income countries including France, Spain, the United States (US) and Canada, ii) describe spatial patterns, and iii) identify vulnerable population sub-groups for UNFR CO poisoning.

## 2 Material and methods

### 2.1 Data sources

#### 2.1.1 Hospital admission data

We extracted data on all non-elective CO poisoning hospital admissions registered in England between 2002 and 2016 from the Hospital Episode Statistics (HES) inpatient dataset (The NHS Information Centre for Health and Social Care, 2018). HES contains detailed information on any admission at a National Health Service (NHS) hospital – i.e. public hospital – in England, including private patients treated in NHS hospitals. Hospital stays are broken down in periods of care under different consultants, referred to as ‘episodes’. The order of episodes reflects the relevance of events in relation to the reason of hospitalization. Each episode contains a primary diagnosis and up to 19 secondary diagnostic fields, coded based on the International Classification of Disease 10th edition (ICD-10) (WHO, 2010). We included hospital records with any mention of unintentional toxic effects of CO (ICD-10: T58 + X47), excluding fire-related hospital admission (ICD-10: X00-X09; T20-T32 or Y26). Nonetheless, other causes of CO poisoning (Appendix Methods A1 & Table A1) were examined for context (Table 1). We considered both primary and secondary diagnostic fields of a first episode. Multiple admissions by the same patient were retained as exposure to the same emission source is plausible. Data on UNFR CO poisoning admissions were obtained from other high-income countries for which data could be accessed freely online (Canada) or through collaborations (US, France and Spain) (Table A2).

**Table 1:** Number of CO-related hospital admissions in England between 2002 and 2016 by sex and cause of poisoning. Fire-related cases were excluded.

CO poisoning type	Male		Female		Total	
	n (%)	Annual average (n/year)^a^	n (%)	Annual average (n/year)^a^	n (%)	Annual average (n/year)^a^
Unintentional (UNFR)	1782 (52.5)	118.8/year	1617 (47.5)	107.8/year	3399 (100)	226.6/year
Intentional	2169 (80.9)	144.6/year	515 (19.1)	34.3/year	2684 (100)	178.9/year
Unknown	332 (57.3)	22.1/year	228 (42.7)	15.2/year	556 (100)	37.1/year
Total	4283 (64.5)	285.5/year	2360 (35.5)	157.3/year	6643 (100)	442.9/year

a Number of cases per year calculated as follows: N_2002–2016_/15_years_.

#### 2.1.2 Individual and area-level indicators of risk

Individual characteristics including sex, age, place of residence (postcode) and date of diagnosis were obtained from the HES hospital records. Individual hospital records were spatially assigned to Middle Layer Super Output Areas (MSOA, 1500 population average), based on the residential postcode at diagnosis. MSOAs are administrative area units for England defined by ONS (Office of National Statistics (ONS), 2009). We considered them to represent the optimum compromise between spatial resolution and statistical power for the presented analyses. Forty-three hospital records had missing information on residential postcode and thus, were excluded from the spatial analysis.

Area-level indicators included: deprivation, rural-urban classification and ethnic composition. For deprivation, we chose the Carstairs Index as it provides a small-area composite measure based on four deprivation indicators: unemployment, household overcrowding, no car ownership and social class (Carstairs and Morris, 1989). Carstairs scores were computed using 2011 census data, standardized at MSOA level and categorised into quintiles for the analysis. For rurality, MSOAs were classified as urban if their constituent Census Output Areas (COA), the smallest statistical area units available for England (29) were predominantly classed as urban (>10,000 people) based on the ONS Rural/Urban Classification 2011 (Office of National Statistics, 2011). Ethnic composition was defined as the proportions of Asians and Blacks below or equal to: i) the national average; ii) 2-fold the national average; and iii) 6-fold the national average (Appendix Methods A2). Data on overall non-white population (i.e. Asian, Black, mixed ethnic groups and other ethnic groups) were also used for a sub-analysis with a similar approach for the cut-offs, here derived from the national non-white population proportion.

Mid-year population estimates stratified by sex, age group, year and geography were obtained from the ONS (Office of National Statistics, 2016).

### 2.2 Statistical analyses

#### 2.2.1 Temporal trends

Age-standardized rate (ASR) trends were studied using joinpoint analysis, which identify change points in trends and estimates the regression function for the segments (Surveillance Research Program National Cancer Institute, 2018). We modelled the ASRs as log-linear piece-wise functions of time where the slope of each of the segment is the annual percentage change (APC). Significance was set at p < 0.05 and tested using the Monte Carlo Permutation method. We tested up to three joinpoints and assessed the model fit and the numbers of joinpoints with the Bayesian Information Criterion (BIC). We also analysed temporal trends for Crude Rates (CR) using sex-specific hospital admission counts per year modelled as log-linear piece-wise functions of time with Poisson variance and a log-population offset (Kim et al., 2000). All joinpoint analyses were conducted using the Joinpoint Trend Analysis Software Version 4.6.0 of the Surveillance Research Program of the National Cancer Institute (Surveillance Research Program National Cancer Institute, 2018). Results for England were compared to those in Canada, France, Spain and the US. All country-specific rates were age-standardized using the Canadian 1991 standard population. Data collected extended from 1995 to 2016, with the range of available years varying for each country (Table A3).

#### 2.2.2 Spatial variability

To evaluate spatial variability at the regional level, we estimated ASRs for the nine Government Office Region (GOF) across England: East Midlands, East of England, Greater London, North East England, North West England, South East England, South West England, West Midlands and Yorkshire and the Humber (Fisher et al., 2014), based on their postcode at diagnosis. In addition, small-area spatial patterns of relative risk (RR) were analysed using the Besag, York and Mollié (BYM) model (Besag et al., 1991) which is a generalized linear mixed effects model that accounts for both spatially unstructured and spatially structured random effects, the latter modelled by an intrinsic conditional auto-regressive (CAR) prior.

#### 2.2.3 Sub-groups at risk

Overall and sex-specific crude rates (CRs), and ASRs, using the WHO standard population, 2000–2025 (Ahmad et al., 2001), were estimate for each area-level indicator category. The ASR 95% confidence intervals (95% CI) were estimated according to the method proposed by Tiwari and colleagues (Tiwari et al., 2006). Age-specific rates were also estimated by broad age groups (i.e. <10; 10–24; 25–39; 40–54; 55–69; 70–84, and ≥85 years old). All rates are given per 100,000 inhabitants.

To evaluate the independent effect of each area-level indicator on the age- and sex-standardized risk of UNFR CO poisoning hospitalization, we fitted a multivariate Poisson regression with random structured and unstructured random effects, following the BYM model (Besag et al., 1991) with a spatial unstructured and spatial structured random effects (Appendix Methods A3). Results are reported as posterior smoothed residual relative risk (RR) estimates for each covariate category and its correspondent 95% credible intervals (95% CrI). Integrated nested Laplace approximations (INLAs) were applied as a tool for Bayesian inference using the *R*-INLA package (Rue, 2017). The proportion of non-whites was used, instead of the proportion of Asians and Blacks, in a sensitivity analysis (Table A3).

## 3 Results

We identified 6643 hospital admissions for CO poisoning in England between 2002 and 2016, excluding fire-related cases (n = 408; 5.9%) (Fig. A1). Of these, 1782 (52.5%) were unintentional, which represented 47.5% (n = 1617) and 52.5% (n = 1782) of female and male hospitalizations, respectively (p = 0.091) (Table 1). Overall, this is equivalent on average to 227 UNFR CO poisoning hospital admissions per year (min_2013_ = 166; max_2010_ = 326) (Table 1).

### 3.1 Temporal trends

**Fig. 1:**
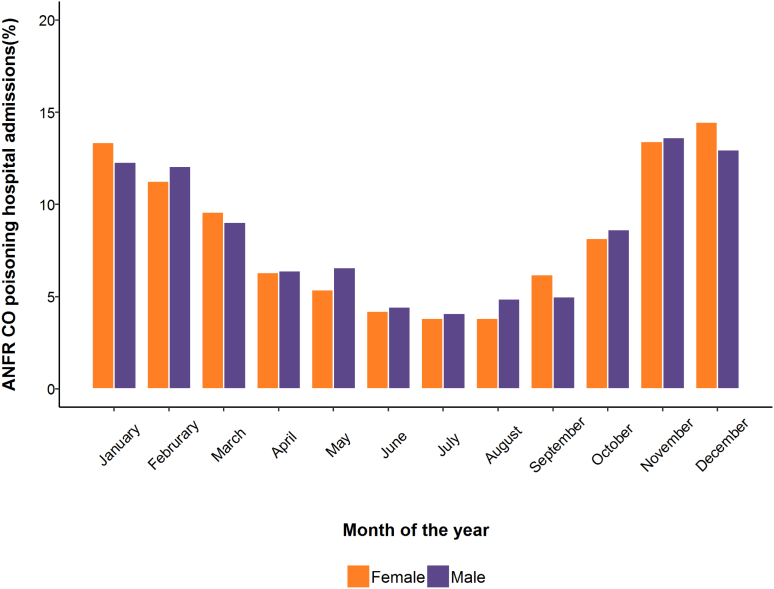
Percentage of ANFR CO poisoning hospital admissions among males (purple) and females (orange) by calendar month, England, 2002–2016. (For interpretation of the references to colour in this figure legend, the reader is referred to the web version of this article.)

**Fig. 2:**
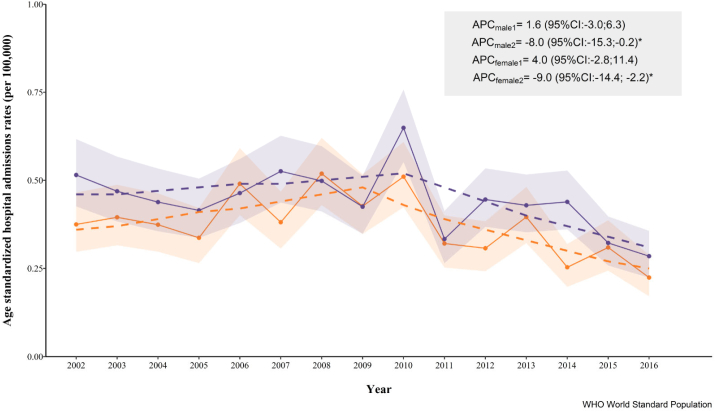
Age-standardized rates (per 100,000 inhab.) of ANFR CO poisoning among male (purple) and females (orange) in England (2002–2016), shadowed areas showing the 95% confidence intervals. In dashed lines, the fitted piecewise linear regression with a joinpoint for females in 2010 and males 2009. The average percentage of change (APC) for each segment with its 95% confidence intervals (CI) is also provided. Rates were age-standardized using the WHO standard population 2000–2025. (For interpretation of the references to colour in this figure legend, the reader is referred to the web version of this article.)

There was clear seasonality in the admissions, with the largest proportion of hospitalisations occurring during winter months (November to February) (Fig. 1). The average annual ASR was 0.44 for males and 0.37 for females, with a peak in 2010 (males: 0.65, females: 0.51) which coincided with the inflection point detected by the Joinpoint analysis (Fig. 2). The trend analysis only suggested a statistically significant decrease for both male and females (−8.0% and −9.0%, respectively) beyond that inflection. Similar results were observed for CR (Fig. A2).

**Fig. 3:**
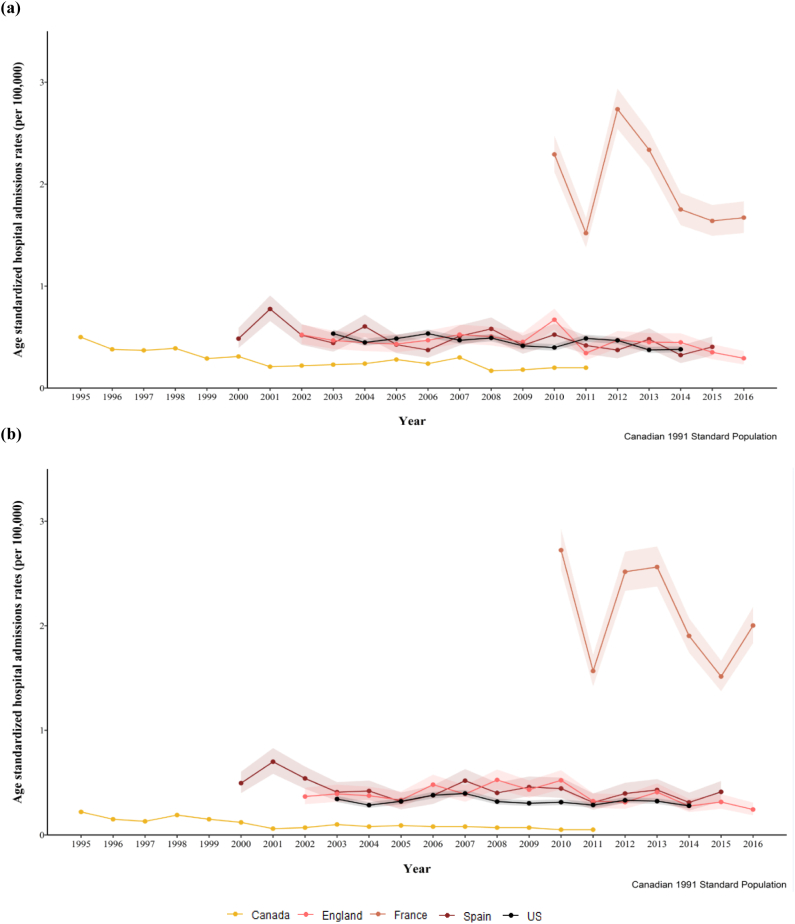
Age-standardized rates of ANFR CO poisoning hospital admissions (per 100,000 inhab.) in England (light pink), France (dark orange), US (black) and Canada (yellow) and Spain (maroon) for (a) males and (b) females. Rates age-standardized using the Canadian 1991 standard population. (For interpretation of the references to colour in this figure legend, the reader is referred to the web version of this article.)

A comparison of rates and temporal trends for the five countries considered is presented in Fig. 3. Rates in Spain (2000–2015), which suggested a slightly negative trend overall, were similar to those found in England. US rates, which remained unchanged between 2003 and 2013, were slightly lower than those reported in England. Canadian rates were consistently lower than those reported in the other countries considered. In France (2010–2015), there was no clear trend and rates were three- to four-fold higher than in England.

### 3.2 Spatial variability

**Fig. 4:**
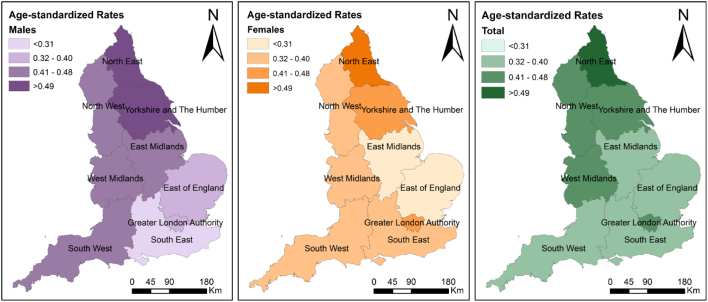
Age-standardized rates per 100,000 population per Governmental Official Region for males (left), females (middle), and total (right).

**Fig. 5:**
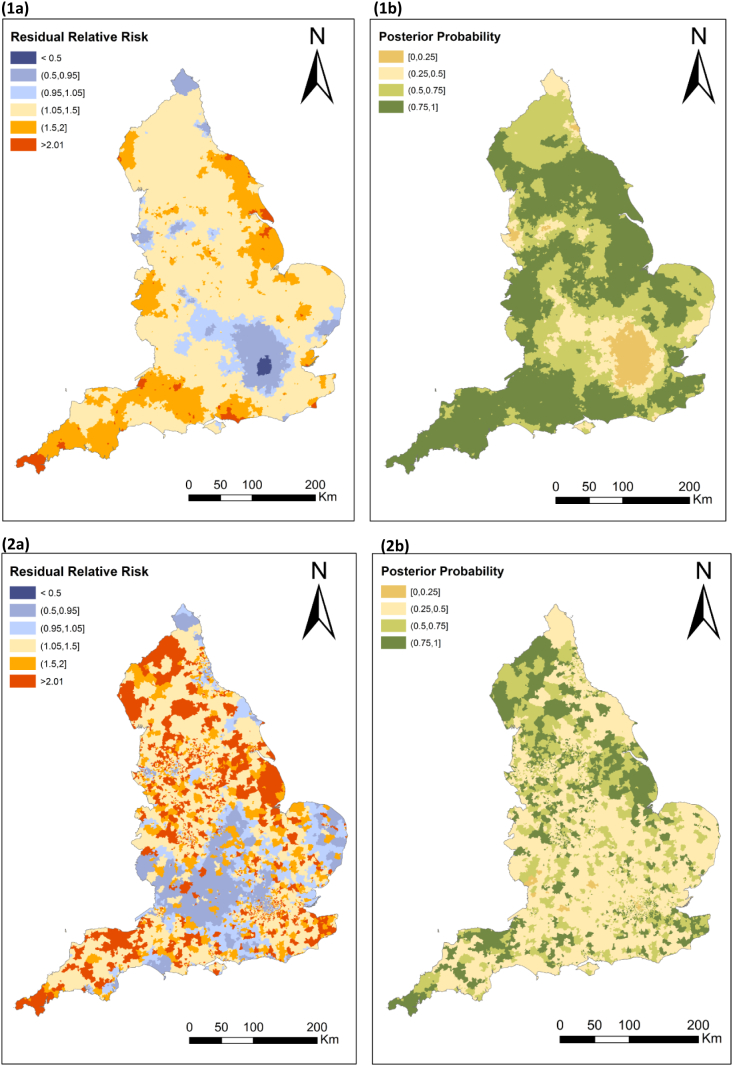
(1) Unadjusted and (2) adjusted estimates of the (a) smoothed residual relative risk and (b) posterior probability of UNFR CO poisoning hospital admission in England, 2002–2016. The model used age and sex standardized rates.

Across the nine GORs, the highest ASRs were found in the North East (ASR = 0.55, 95% CI: 0.47; 0.63) and Yorkshire and the Humber (ASR = 0.48; 95% CI: 0.43; 0.53) and the lowest in the East (ASR = 0.32; 95% CI: 0.28; 0.36) and the South East of England (ASR = 0.33, 95% CI: 0.30; 0.37) (Table 2, Fig. 5). Rates were lower among females, with the exception of the Greater London Authority and the South East where higher rates were found among females (Fig. 4). The unadjusted small area analysis with spatial smoothing (Fig. 5.1a) had an increased risk between 1.05 and 1.5 across all England with the exception of the Greater London Authority and surrounding areas, yet those associated to low confidence (Fig. 5.1b). Our maps also highlighted high-risk pockets across the South West, and in the coastline across the South East, East Midlands and North East of England. Spatial patterns appeared more fragmented after adjustments for area-level deprivation, rurality and ethnic composition (Fig. 5.2a and b).

**Table 2:** Crude (CR) and age-standardized (ASR) rates of ANFR hospital admissions in England (2002–2016) by population characteristics. CI: confidence interval.

	Total				Male				Female			
	N	CR	ASR	(95% CI)	N	CR	ASR	(95% CI)	N	CR	ASR	(95% CI)
Total	3399	0.43	0.41	(0.39;0.42)	1782	0.46	0.44	(0.42;0.46)	1617	0.41	0.37	(0.35;0.39)
Age groups (years old)												
<10	476	0.51	–		253	0.53	–		223	0.49	–	
10 to 24	476	0.32	–		237	0.32	–		239	0.33	–	
25 to 39	670	0.41	–		379	0.47	–		291	0.36	–	
40 to 54	600	0.37	–		346	0.43	–		254	0.31	–	
55 to 69	419	0.33	–		236	0.38	–		183	0.28	–	
70 to 84	486	0.65	–		218	0.65	–		268	0.65	–	
>85	272	1.61	–		113	2.09	–		159	1.38	–	
Governmental official regions^a^												
East of England	313	0.35	0.32	(0.28;0.36)	185	0.43	0.40	(0.34;0.46)	128	0.28	0.25	(0.20;0.30)
East midlands	288	0.42	0.39	(0.34;0.44)	169	0.51	0.48	(0.41;0.57)	119	0.34	0.29	(0.23;0.36)
London	513	0.42	0.41	(0.37;0.45)	236	0.40	0.40	(0.34;0.45)	277	0.43	0.43	(0.38;0.49)
North east	193	0.49	0.55	(0.47;0.63)	102	0.54	0.58	(0.47;0.71)	91	0.44	0.53	(0.42;0.66)
North west	483	0.45	0.42	(0.38;0.46)	246	0.48	0.46	(0.40;0.52)	237	0.43	0.38	(0.33;0.44)
South east	471	0.36	0.33	(0.30;0.37)	209	0.33	0.31	(0.27;0.36)	262	0.39	0.36	(0.32;0.42)
South west	336	0.42	0.38	(0.34;0.43)	189	0.49	0.47	(0.40;0.54)	147	0.35	0.31	(0.25;0.37)
West midlands	362	0.43	0.42	(0.37;0.46)	193	0.47	0.47	(0.41;0.55)	169	0.39	0.36	(0.30;0.42)
Yorkshire and the Humber	385	0.48	0.48	(0.43;0.53)	216	0.56	0.56	(0.49;0.64)	169	0.41	0.41	(0.34;0.48)
Rural/urban classification^a^												
Rural	640	0.48	0.46	(0.42;0.50)	364	0.55	0.51	(0.46;0.58)	276	0.41	0.41	(0.35;0.47)
Urban	2704	0.41	0.39	(0.37;0.40)	1381	0.43	0.41	(0.39;0.43)	1323	0.39	0.36	(0.34;0.38)
Carstairs Index^a^												
Q1 - least deprived	565	0.35	0.33	(0.30;0.36)	302	0.38	0.35	(0.31;0.40)	263	0.32	0.30	(0.26;0.35)
Q2	537	0.34	0.30	(0.27;0.33)	283	0.36	0.33	(0.29;0.38)	254	0.31	0.27	(0.23;0.31)
Q3	664	0.43	0.40	(0.37;0.43)	342	0.46	0.44	(0.39;0.49)	322	0.41	0.36	(0.32;0.41)
Q4	696	0.47	0.44	(0.41;0.48)	353	0.48	0.47	(0.42;0.53)	343	0.45	0.41	(0.37;0.46)
Q5 - most deprived	882	0.51	0.50	(0.47;0.54)	465	0.55	0.54	(0.49;0.59)	417	0.48	0.47	(0.42;0.51)
Black population (%)^a^												
<3.5%	2567	0.42	0.40	(0.38;0.42)	1368	0.46	0.44	(0.42;0.47)	1199	0.39	0.36	(0.33;0.38)
3.5–7%	237	0.32	0.30	(0.26;0.34)	112	0.30	0.29	(0.24;0.36)	125	0.33	0.30	(0.24;0.36)
7–21%	376	0.44	0.44	(0.39;0.49)	186	0.43	0.44	(0.38;0.51)	190	0.44	0.44	(0.38;0.51)
>21%	164	0.56	0.57	(0.48;0.67)	79	0.55	0.56	(0.44;0.70)	85	0.58	0.58	(0.46;0.72)
Asian population (%)^a^												
<7.8%	2483	0.42	0.40	(0.38;0.42)	1316	0.46	0.44	(0.41;0.46)	1167	0.39	0.36	(0.34;0.39)
7.8–15%	378	0.36	0.33	(0.30;0.37)	195	0.38	0.37	(0.31;0.42)	183	0.35	0.30	(0.25;0.35)
15–47%	337	0.42	0.41	(0.37;0.46)	157	0.39	0.39	(0.33;0.46)	180	0.45	0.43	(0.37;0.50)
>47%	146	0.59	0.61	(0.51;0.71)	77	0.62	0.64	(0.50;0.80)	69	0.57	0.57	(0.44;0.73)

a 43 hospital admissions excluded due to missing geographical information.

### 3.3 Sub-groups at risk

The highest age-specific CRs were found among those aged over 85 years (1.61, Table 2). CRs among children aged <10 years old were slightly higher (0.51) than those found in older children, 10 to 24 years old (0.32) and young adult groups, 25 to 29 years old (0.41). We found higher ASRs in rural areas (0.46) compared to urban areas (0.39). Rates in areas with the largest proportion of Blacks and Asians were higher (0.57 and 0.61, respectively) than in those with a lower proportion than the national average (0.42 and 0.42, respectively). The most deprived areas had a 45% increased rate as compared to the least deprived (0.51 and 0.35, respectively). These patterns were observed both overall and by gender. ASRs were slightly higher among males than females, 0.44 and 0.37, respectively.

In our multivariate model (Table 3), we found a reduced risk of UNFR CO poisoning hospitalization in urban areas (RR = 0.69, 95% CrI: 0.67; 0.80). We observed an association between risk and area-level deprivation, with the most deprived areas showing a 1.7-fold increase (RR: 1.76, 95% CrI: 1.66; 2.10). in relation to ethnicity, areas with the highest proportion of Asians had the highest risk (RR = 1.15, 95% CrI: 1.04; 1.50). Areas with >21% of Blacks had a 1.35 (95% CrI: 1.20; 1.80) increased risk compared to areas with <3.5% (average England). When the model was fitted using the proportion of non-Whites (Table A3), the effects of rurality and deprivation remained, although no effect was seen for areas with a higher proportion of non-White population (1.04; 95% CrI: 0.96; 1.29).

**Table 3:** Estimates (relative risk, RR) of the fully adjusted model for ANFR CO poisoning hospital admission in England, 2002–2016^a^. The model used age and sex standardized rates.

	RR	(95% CrI)
Rural/urban classification		
Rural	1	
Urban	0.69	(0.67;0.80)
Carstairs index		
Q1 - least deprived	1	
Q2	0.98	(0.93;1.13)
Q3	1.35	(1.28;1.56)
Q4	1.55	(1.46;1.81)
Q5 - most deprived	1.77	(1.66;2.10)
Asian population (%)		
<7.8%	1	
7.8–15%	0.93	(0.89;1.09)
15–47%	0.94	(0.89;1.13)
>47%	1.15	(1.03;1.49)
Black population (%)		
<3.5%	1	
3.5–7%	0.76	(0.71;0.91)
7–21%	1.00	(0.92;1.21)
>21%	1.35	(1.20;1.80)

RR, Relative Risk; 95% CrI, 95% Credible Intervals.

a 43 hospital admissions excluded due to missing geographical information.

## 4 Discussion

Our study combined 15-years of nationally representative health data on UNFR CO poisoning hospitalizations with a range of individual and area-level characteristics, to provide detailed spatio-temporal epidemiological evidence for England, and to identify sub-groups at higher risk to guide future public health interventions.

### 4.1 Temporal trends

As in previously published studies (Khadem-Rezaiyan and Afshari, 2016; Lavigne et al., 2015; Wilson et al., 1998; Iqbal et al., 2012a; Graber et al., 2007), our data indicated a clear seasonality of CO poisoning, with admissions being more common during colder months. Lower temperatures and especially extreme conditions are strongly associated with higher consumption of gas and electricity, increased heating appliances usage and more time spent indoors (Verrier et al., 2010; Peck, 2011). This is also reflected in temporal trends observed over our study period. The highest rates of UNFR CO poisoning hospitalizations in England in 2010 coincided with the coldest winter recorded since 1979 with a mean winter temperature 2 °C lower than the average temperature recorded during 1971–2000 (Prior and Kendon, 2011). This correlated with a peak in energy consumption for home heating (Department for Business Energy and Industrial Strategy, 2017), which is associated with an increased likelihood of CO poisoning (Lutterloh et al., 2011; Gilles et al., 2008).

Our joinpoint analysis indicated an inflection point around 2010 in the ASR of UNFR CO poisoning hospitalizations both for females and males. Although this shift in the trends could have been driven by the 2010's cold winter, it seems more likely to reflect the impact of a new regulation, implemented in 2010, making the installation of a CO detector mandatory in all new buildings with solid fuel appliances (Secretary of State E and W, 2010). This legislation, together with a gradual increase in the awareness, prevention campaigns and/or media coverage of CO poisoning fatal cases may have contributed to the observed decreasing trends. This regulation was extended to private sector rental properties in 2015 (The Smoke and Carbon Monoxide Alarm (England) Regulations 2015, 2015), but our study period did not allow to assess whether this legislation had further impact.

Our international comparison showed substantial variation between countries, with the lowest rates across the reporting periods observed in Canada and the highest in France and the US. This international variability has previously been reported for both CO-related hospital admissions and deaths across different countries in Europe (Braubach et al., 2013). Multiple factors including climate, data recording differences, socio-demographic characteristics, housing stock characteristics, energy efficiency, and legislation, to mention a few, may have contributed. Standardized regulations, such as those implemented across the European Union, could help facilitating such comparisons.

### 4.2 Spatial variability

We found substantial regional variations across England. According to Energy Performance Certificate (EPC) data, areas in the South West and North of England have a higher percentage of oil- and wood-fired primary heating appliances (Shrubsole et al., 2017). After adjusting for rurality, deprivation and ethnicity, the spatial patterns became more inconsistent with more pockets of high and low risk across England, which suggests that the spatial variation of UNFR CO poisoning hospital admissions is substantially influenced by the distribution of area-level characteristics considered. Cluster analyses may help identify areas which are particularly at risk.

### 4.3 Sub-groups at risk

Our study found males, children and the elderly, and individuals living in rural, deprived or ethnically diverse areas to be at a higher risk of UNFR CO poisoning hospital admission. Higher rates of UNFR CO poisoning hospitalizations observed in males are consistent with previous studies on CO poisoning (Lavigne et al., 2015; Iqbal et al., 2012b) or poisoning in general (Peiris-John et al., 2014). Although reasons behind these differences remain unclear, it has been suggested that this could be related to the use of fuel-burning appliances (Bosch, 2013; Iqbal et al., 2010), occupational exposure (WHO, 2004), time spent at home (Lavigne et al., 2015; Iqbal et al., 2012b; Clifton et al., 2001; Sircar et al., 2015), or the likelihood of being hospitalized (Iqbal et al., 2010, Iqbal et al., 2012a). The observed bi-modal distribution across age (<10 and >85 years old), similar to that reported elsewhere (Health and Safety Executive, 2019), could be explained by the greater susceptibility of the elderly and children to the manifestation of CO poisoning symptoms (Smollin and Olson, 2008; WHO, 2004). Therefore, children and the elderly could be acting as early indicators of CO exposure in the home. In addition, the elderly spend greater amount of time spent at home (Lavigne et al., 2015; Iqbal et al., 2012b; Clifton et al., 2001; Spalt et al., 2016), and they tend to reside in old households with heating appliances less likely to be regularly checked (Braubach et al., 2013; WHO, 2004).

Rural areas had an excess risk compared to urban areas, which remained even after adjusting for area deprivation and ethnic composition. Similar results have been reported in the US (Iqbal et al., 2012b; Sircar et al., 2015) and Romania (Nistor et al., 2018). According to data from the English Housing Survey and EPC, solid, oil and electric fuel appliances are more common in English rural areas whereas other fuel types (gas or electric) are often used in apartments, the most common building type in urban areas. Furthermore, in a pilot study assessing low income households in England, rural households with non-gas appliances were found to be linked to older and riskier boiler types (National Energy Action, 2017).

Areas with a high prevalence of Asians and Blacks have an increased risk of UNFR CO poisoning hospitalization, independently of socio-economic status. The lack of effect observed when using the proportion of non-Whites instead, seems to suggest the existence of cultural practices (e.g. choice of cooking and/or heating appliance type) rooted in the Asian and Black communities may influence their exposure to CO. An earlier study in the US reported an increased prevalence of indoor burning of charcoal briquettes among Asian populations (Ralston et al., 2012). More research is needed to confirm these results and provide a more detailed insight in its drivers.

Our results in relation to deprivation contrasts with earlier evidence for England (1988–1994) (Wilson et al., 1998) and (2001−2010) (Ghosh et al., 2016) where no linear relations were observed. However, these studies considered deprivation alone as opposed to our multivariate model which included ethnicity composition, rural/urban classification, age and sex as well as spatial structure. A widening in the health inequality gap between 2001 and 2016 (Bennett et al., 2018) could also explain these differences. The increasing risk of UNFR CO poisoning hospitalization by area-level deprivation quintile could be related to a higher prevalence of smoking, poor housing conditions and poor maintenance of heating and cooking appliances. Previous studies have reported that home exposures and malfunctioning heating systems represent the majority of UNFR CO poisoning cases (Smollin and Olson, 2008; Penney et al., 2010; Shrubsole et al., 2017; Clifton et al., 2001; Croxford et al., 2006). According to a UK survey, 40% of low-income respondents stated that they faced the choice between ‘heating or eating’ dilemma (Cooper et al., 2015). This may lead to risk behaviours including reduction of ventilation, lack of maintenance of appliances, and use of old, poorer quality supplementary heating appliances to reduce their central heating costs (Ormandy and Ezratty, 2012). According to a report produced by the National Energy Action in 2017, households reporting stress and anxiety about energy affordability were more likely to have peaks of CO levels >10 ppm and for these to be longer (National Energy Action, 2017). Furthermore, a vulnerability differential is plausible as multiple risk factors tend to cluster in low socioeconomic groups (Feng and Astell-Burt, 2013; Halonen et al., 2012; Hussein et al., 2018), which may trigger biological synergism between existing conditions and the effects of CO exposure. Such clustering of risk factors in deprived groups has been widely studied for some diseases such as cardiovascular disease and diabetes (Kivimäki et al., 2007; Sharma et al., 2004).

### 4.4 Study strengths and limitations

This analysis of the risk of UNFR CO poisoning hospitalization in various population sub-groups in England is based on a nationally representative number of cases of CO poisoning, combined with a wide range of individual and area-level characteristics. However, our study only reflects severe cases that require hospitalisation; which may be affected by misdiagnosis, a prevalent issue in CO poisoning (Clarke et al., 2012) that contributes to its underestimation, and although our approach provides valuable insights into regional and local differences, it is subject to the ecological bias as the differences in risk shown apply to populations rather than individuals.

### 4.5 Public health implications

Our study provides robust evidence that CO poisoning remains as neglected preventable cause of morbidity in England despite their consistent decrease since 2010. Our comparison with other countries suggested that further reductions in morbidity and mortality of CO poisoning should be possible. This is relevant as the economic burden of UNFR CO poisoning from direct care costs, long-term reductions on the earning potential of the population or death, can be substantial (Hampson, 2016; Ran et al., 2018).

Finally, the evidence provided here on sub-groups at risk should be considered when planning and implementing targeted preventive measures, such as designing information/awareness campaigns targeted to sub-groups at risk, distributing free CO alarms, and implementing improvements in the housing sector to alleviate fuel poverty and to reduce the risk of CO poisoning among deprived populations.

## 5 Conclusion

This study highlights the urgent need to address the inequalities in the risk of CO poisoning and provides information on population groups at risk which can be used to develop more adequate and targeted measures.

## CRediT authorship contribution statement

**Aina Roca-Barceló:**Conceptualization, Methodology, Formal analysis, Investigation, Writing - original draft, Writing - review & editing.**Helen Crabbe:**Writing - review & editing.**Rebecca Ghosh:**Writing - review & editing.**Anna Freni-Sterrantino:**Formal analysis, Writing - review & editing.**Tony Fletcher:**Writing - review & editing.**Giovanni Leonardi:**Conceptualization, Writing - review & editing.**Courtney Hoge:**Resources, Writing - review & editing.**Anna L. Hansell:**Conceptualization, Writing - review & editing.**Frédéric B. Piel:**Conceptualization, Methodology, Writing - review & editing, Supervision.

## Declaration of competing interest

The authors declare that they have no conflict of interest.

## Appendix A Supplementary data

Supplementary material Image 1
