# Supplementary material for: Temporal trends and demographic risk factors for hospital admissions due to carbon monoxide poisoning in England
Source: Prev Med. 2020 Jul;136:106104. doi: 10.1016/j.ypmed.2020.106104 (PMC7262581; doi:10.1016/j.ypmed.2020.106104)

# Appendix

Contents

[Appendix - Methods 2](#_Toc38987414)

[A1. Classification of CO poisoning 2](#_Toc38987415)

[A2. Ethnicity indicator 2](#_Toc38987416)

[A3. Model description 2](#_Toc38987417)

[Appendix - Tables 4](#_Toc38987418)

[Table A1. Included ICD-10 codes and description. 4](#_Toc38987419)

[Table A2. Summary information on published and obtained data for ANFR CO poisoning hospital admissions by country. Rates have been standardized using the Canadian 1991 standard population unless otherwise specified. 5](#_Toc38987420)

[Table A3. Estimates of the relative risk of ANFR CO poisoning hospital admission in England, 2002-2016*, in a fully adjusted model. The model used age and sex standardized rates. 6](#_Toc38987421)

[Appendix- Figures 7](#_Toc38987422)

[Figure A1. Flowchart illustrating the selection criteria used to classify carbon monoxide poisoning hospital admission data for England (2002-2016). NFR, non-fire related. 7](#_Toc38987423)

[Figure A2. Crude rates of ANFR hospital admissions among males (purple) and females (orange) by sex in England, 2002-2016. Solid lines show the piecewise linear regression with a joinpoint in 2010. 8](#_Toc38987424)

# Appendix - Methods

## A1. Classification of CO poisoning

Admissions were classified based on the reported intention as being (i) unintentional (ICD-10: T58 + X47); (ii) intentional (ICD-10: T58 + X67) or, (iii) of unknown intent (ICD-10: T58 + Y17 and admissions where no causal code was specified). All fire-related hospital admission (ICD-10: X00-X09; T20-T32 or Y26) were excluded. Only UNFR CO poisoning hospital admissions are reported here. Results of a parallel analysis of intentional UNFR admissions will be reported separately. *Supplementary Table A1* summarizes the included ICD-10 codes and their descriptions.

## A2. Ethnicity indicator

MSOA-level ethnic composition was defined as the proportion of Asian (census term “Asian or Asian British,” which includes “Indian,” “Pakistani,” “Bangladeshi”, “Chinese” and “Other Asian”) and Black population (census term “Black or Black British,” which includes “Black Caribbean,” “Black African,” and “Other Black”) per MSOA, which are the two major non-white ethnic groups in England. We used the following cut-off points: the national average (%) of Asian and Black population for England at MSOA level (7.8%, 3.5%), 2-fold the national average (15%, 7%), and 6-fold the national average (47%, 21%), respectively. We used those areas where the proportion was less than or equal to the national average (%) as the reference category. Furthermore, we explored the overall non-white population composition which included Asian and Black populations, as well as mixed ethnic groups (census term “Mixed/Multiple ethnic groups”, which includes “White and Black Caribbean”, “White and Black African”, “White and Asian” and “Any other Mixed/Multiple ethnic backgrounds”) and other ethnic group populations (census term “Other ethnic group”, which includes “Arab” and “Any other ethnic group”). We classified them using the following cut-off points: 15% (the national average), 30% (2-fold the national average), 50% (majority of non-white ethnicities).

## A3. Model description

To evaluate the independent effect of area-level deprivation, ethnic composition and rural-urban classification on the age and sex standardized risk of UNFR CO poisoning hospitalization, we fitted a Poisson regression with random structured and unstructured effects as conditional autoregressive spatial model, as developed by Besag, York and Mollié (BYM) [35]. The model included two random effects terms: (i) a structured component, to capture spatial dependency due to shared characteristics with neighbouring areas (i.e. areas nearby are more similar than those far apart), and (ii) an unstructured component (heterogeneity), which is explained by unobserved variables. The neighbouring structure was defined by adjacency of MSOA boundaries. The model equation is presented below (Eq. A1).

**Eq. A1.** Bayesian conditional autoregressive spatial equation for the fully adjusted main model for UNFR CO poisoning hospital admissions.

| Obs_i_∼Poisson(Exp_i_ λ_i_)  log(λ_i_) = α + 𝛽_1_Carstairs_i_ + 𝛽_2_Asian_𝑖_ + 𝛽_3_Black_i_+ 𝛽_4_Rural/Urban_i_ + h_i_ + b_i_ |  |
| --- | --- |

Where:

Obs_i_ is the number of hospital admissions in MSOAi

Po is Poisson distribution

Exp_i_ are the expected number of UNFR CO poisoning hospital admissions

*λ* _i_ is the relative risk in MSOAi

α is the intercept

𝛽_1_Carstairs_i_ is the regression coefficient for the Carstairs quintile for MSOA_i_

𝛽_2_Asian_𝑖_ is the regression coefficient for the Asian composition at MSOA_i_

𝛽_3_Black_𝑖_ is the regression coefficient for the Black composition at MSOA_i_

𝛽_4_Rural/Urban_𝑖_ is the regression coefficient for the rural/urban classification for MSOA_i_

h_i_ is the MSOA heterogeneity term (unstructured component)

b_i_ is the spatial term (structured component).

# Appendix - Tables

| **Table A1**. Included ICD-10 codes and description | |
| --- | --- |
| **ICD-10 code** | **Description** |
| T58.X | Toxic effect of carbon monoxide |
| **CO poisoning by cause** | |
| T58 + X47 | Unintentional poisoning by and exposure to other gases and vapours |
| T58 + X67 | Intentional self-poisoning by and exposure to organic solvents and halogenated hydrocarbons and their vapours |
| T58 + Y17 | Poisoning by and exposure to other gases and vapours, undetermined intent |
| **Fire-related exposures** | |
| X00-X09 | Exposure to smoke, fire and flames |
| T20-T32 | Burns and corrosions |
| Y26 | Exposure to smoke, fire and flames, undetermined intent |

## **Table A2**. Summary information on published and obtained data for ANFR CO poisoning hospital admissions by country. Rates have been standardized using the Canadian 1991 standard population unless otherwise specified

| **Country** |  | **Reporting period** | **Reporting years** | **Total hosp. admissions** | **Average annual rates*** | **Lowest admission rate *(year)*** | **Highest admission rate *(year)*** | |
| --- | --- | --- | --- | --- | --- | --- | --- | --- |
| England^a^ | *Male* | 2001-2010 | 9 | 1,617 | 0.41 | 0.33 *(2016)* | 0.68 *(2010)* | |
|  | *Female* |  |  | 1,782 | 0.46 | 0.28 *(2016)* | 0.55 *(2010)* | |
|  | *Total* |  |  | 3,399 | 0.43 | 0.30 *(2016)* | 0.62 *(2010)* | |
|  |  |  |  |  |  |  |  | |
| Canada ^b^ | *Male* | 1995-2011 | 16 | 1441 | 0.28 | 0.17 *(2008)* | 0.50 *(1995)* | |
|  | *Female* |  |  | 543 | 0.10 | 0.05 *(2010)* | 0.22 *(1995)* | |
|  | *Total* |  |  | 1984 | 0.39 | - | - | |
|  |  |  |  |  |  |  |  | |
| France^c^ | *Male* | 2010-2016 | 6 | 4,155 | 1.92 | 1.50 *(2011)* | 2.62 *(2012)* | |
|  | *Female* |  |  | 4,464 | 1.94 | 1.43 *(2015)* | 2.51 *(2010)* | |
|  | *Total* |  |  | 8,619 | 1.94 | 1.47 *(2011)* | 2.49 *(2012)* | |
|  |  |  |  |  |  |  |  | |
| Spain^d^ | *Male* | 2000-2016 | 16 | 1,670 | 0.47 | 0.30 *(2014)* | 0.72 *(2001)* | |
|  | *Female* |  |  | 1,552 | 0.42 | 0.30 *(2014)* | 0.63 *(2001)* | |
|  | *Total* |  |  | 3,222 | 0.44 | 0.30 *(2014)* | 0.68 *(2001)* | |
|  |  |  |  |  |  |  |  | |
| US^e^ | *Male* | 2003-2014 | 12 | 8,025 | 0.48 | 0.41 *(2013)* | 0.53 *(2003)* | |
|  | *Female* |  |  | 6,273 | 0.36 | 0.30 *(2004)* | 0.37 *(2010)* | |
|  | *Total* |  |  | 14,298 | 0.43 | 0.38 *(2004)* | 0.48 *(2006)* | |
|  |  |  |  |  |  |  |  | |
| *Rates given per 100,000 per year  ¥ Reported period specific for each study.  ¥ ¥ Average computed based on rates for 2010 and 2011 which are the common reported period across all studies  ^a^ Ghosh R et al. (2015) | | | | | | | |  |
| ^b^ ASR directly obtained from Lavigne E et al. (2014) | | | | | | | |  |
| ^c^ Counts provided by the Subdireccion General de Informacion Sanitaria y Evaluacion, Ministerio de Sanidad, Servicios Sociales e Igualdad, Spain. | | | | | | | |  |
| ^d^ Counts provided by the Agence Nationale de Sante Publique, France. | | | | | | | |  |
| ^e^ Counts provided by the National Environmental Public Health Tracking, Centers for Disease Control and Prevention (CDC), USA. | | | | | | | |  |

## **Table A3**. Estimates of the relative risk of ANFR CO poisoning hospital admission in England, 2002-2016*, in a fully adjusted model. The model used age and sex standardized rates

|  |  | **RR** | **(95% CrI)** |
| --- | --- | --- | --- |
| **Rural/Urban Classification** | |  |  |
| Rural |  | 1 |  |
| Urban |  | 0.70 | (0.66; 0.79) |
| **Carstairs Index** | |  |  |
| Q1 - Least deprived | | 1 |  |
| Q2 |  | 0.98 | (0.93;1.13) |
| Q3 |  | 1.34 | (1.27;1.55) |
| Q4 |  | 1.55 | (1.46;1.80) |
| Q5 - Most deprived | | 1.81 | (1.70;2.15) |
| **Non-white population (%)** | |  |  |
| <3.5% |  | 1 |  |
| 3.5-7% |  | 0.84 | (0.79;0.97) |
| 7-21% |  | 0.88 | (0.82;1.07) |
| >21% |  | 1.04 | (0.96;1.29) |
| RR, Relative Risk; 95% CrI, 95% Credible Intervals  *43 hospital admissions excluded due to missing geographical information. | |  |  |

# Appendix- Figures

##

## **Figure A1**. Flowchart illustrating the selection criteria used to classify carbon monoxide poisoning hospital admission data for England (2002-2016). NFR, non-fire related.

**Total (n=7,051)**

Unintentional (n=3,486; 49.5%)

Intentional (n= 2,693; 38.3%)

Unknown (n= 872; 12.1%)

Fire-related hospital admissions excluded

Total (n= 408)

Unintentional (n=87)

Intentional (n=9)

Unknown (n=316)

**NFR Total (n=6,643)**

NFR Unintentional (n= 3,399 51.3%)

NFR Intentional (n= 2,684; 40.5%)

NFR Unknown (n=556; 8.1%)

## **Figure A2**. Crude rates of ANFR hospital admissions among males (purple) and females (orange) by sex in England, 2002-2016. Solid lines show the piecewise linear regression with a joinpoint in 2010


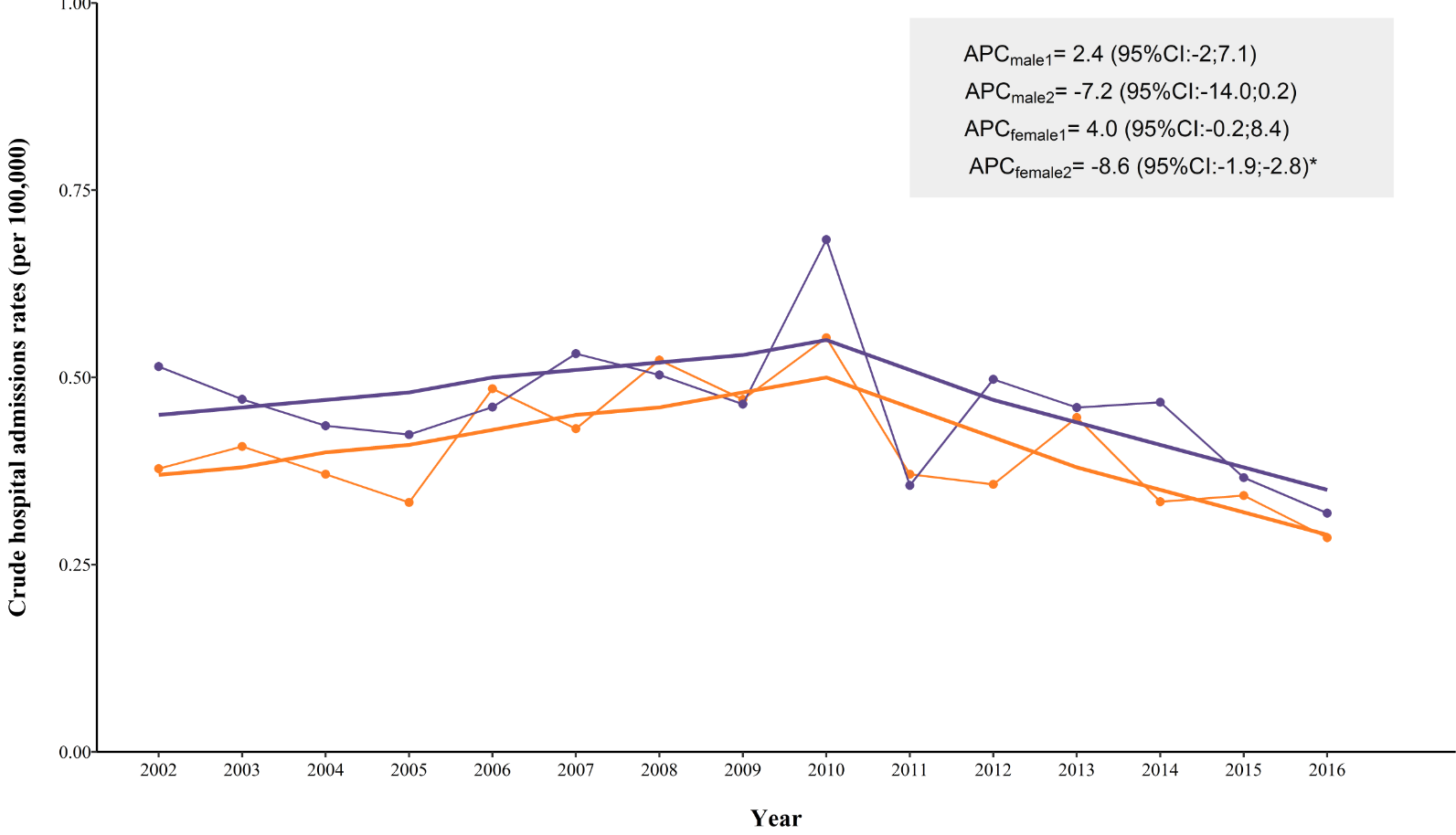

Supplement: Supplementary file 1 — Supplementary material [file mmc1.docx]
